# Static and Dynamic Multiparameter Assessment of Structural Elements Using Chirped Fiber Bragg Gratings

**DOI:** 10.3390/s23041860

**Published:** 2023-02-07

**Authors:** Leandro Macedo, Edson A. Souza, Anselmo Frizera, Maria José Pontes, Carlos Marques, Arnaldo Leal-Junior

**Affiliations:** 1 Graduate Program in Electrical Engineering, Federal University of Espírito Santo, Vitoria 29075-910, Espírito Santo, Brazil; 2 Department of Physics and I3N, University of Aveiro, Campus Universitário de Santiago, 3810-193 Aveiro, Portugal

**Keywords:** optical fiber sensors, chirped fiber Bragg gratings, structural health monitoring, vibration assessment

## Abstract

This paper presents the development, analysis, and application of chirped fiber Bragg gratings (CFBGs) for dynamic and static measurements of beams of different materials in the single-cantilever configuration. In this case, the beams were numerically analyzed using the finite-element method (FEM) for the assessment of the natural frequencies and vibration modes of the beam for the dynamic analysis of the structural element. Furthermore, the static numerical analysis was performed using a load at the free end of the beam, where the maximum strain and its distribution along the beam were analyzed, especially in the region at which the FBG was positioned. The experimental evaluation of the proposed CFBG sensor was performed in static conditions for forces from 0 to 50 N (in 10 N steps) applied at the free end of the beam, whereas the dynamic evaluation was performed by means of positioning an unbalanced motor at the end of the beam, which was excited at 16 Hz, 65 Hz, 100 Hz, and 131 Hz. The results showed the feasibility of the proposed device for the simultaneous assessment of the force and strain distribution along the CFBG region using the wavelength shift and the full-width at half-maximum (FWHM), respectively. In these cases, the determination coefficients of the spectral features as a function of the force and strain distribution were higher than 0.99 in all analyzed cases, where a potential resolution of 0.25 N was obtained on the force assessment. In the dynamic tests, the frequency spectrum of the sensor responses indicated a frequency peak at the excited frequency in all analyzed cases. Therefore, the proposed sensor device is a suitable option to extend the performance of sensors for structural health assessment, since it is possible to simultaneously measure different parameters in dynamic and static conditions using only one sensor device, which, due to its multiplexing capabilities, can be integrated with additional optical fiber sensors for the complete shape reconstruction with millimeter-range spatial resolution.

## 1. Introduction

The performance of civil and mechanical structures is affected over time by several factors such as corrosion, load conditions, and cracks formation [1]. The damage suffered by the structure during service time may lead to critical conditions in operation, decreasing its safety. Furthermore, some types of events not predicted in the project step may result in the collapse of structure [2]. In structural health monitoring (SHM), the data of the structural parameters are acquired by the sensors and are analyzed in order to evaluate the structural performance [3]. SHM provides greater structural control and safety to identify, locate, and evaluate the damage, offering ideal preventive measures to efficiently maintain a structure [4,5].

Optical sensors are capable of measuring several parameters, which are employed in different areas, from biomedical to industrial applications [6]. Optical sensors are intrinsically safe and immune to the corrosion process [7,8]. Due to their light weight and compactness, optical sensors may be easily integrated into the structure or embedded into materials such as concrete and composites, as performed in [9]. In addition, the multiplexing capabilities of the optical fiber sensors, especially the fiber Bragg gratings (FBG), make them useful for distributed structural monitoring [9]. In this case, not only the strain and deflection can be monitored, but also the vibration and acceleration in the structural components.

Since the 1990s, optical fiber accelerometers have been developed [10]. These sensors can be easily embedded in complex structures (such as bridges or highways) for distributed vibration measurement [11,12]. Depending on the application, the design of optical accelerometers must consider at least two parameters: sensitivity and the range of application (based on the mechanical system’s natural frequency) [13]. In addition to the various combinations of these two parameters, new technologies have been developed in the optical accelerometer field to enhance some of the characteristics of these sensors, such as manufacturing [14], sensitivity [15,16], and low cross-sensitivity in different planes [17,18].

In optical fibers, a popular approach for sensing applications is the use of FBGs due to their multiplexing capabilities in conjunction with the possibility of integration into different structures [19]. In addition, different types of refractive index modulation can be used to produce non-uniform gratings [20]. Chirped FBGs (CFBGs) are produced by varying the period of the refractive index modulation along the grating length [21,22,23]. Since the reflected Bragg wavelength is proportional to the effective refractive index and the period of the grating, in CFBGs, each region of the grating reflects a different spectrum [24]. Consequently, the CFBGs’ reflection spectrum is broader than that of uniform FBGs, constituting a cascade of FBGs (each reflecting a narrow spectrum) [21]. Some applications of CFBGs are: the refractive index and temperature sensing [25], force intensity and location assessment [26], damage detection in aerospace structures [27], pulsed mechanical action sensors [28], and for strain [29] and thermal ablation assessment [30]. Such grating devices are capable of detecting the strain [26] and temperature [21] distribution along the grating region, where the CFBGs can also be used in the temperature compensation approaches using a single FBG [31].

This paper presents a CFBG-based sensor embedded into two different beams made from Pinus wood and Nylon 6.0. The Pinus wood beam was used for dynamic experiments with an unbalanced load at the free end of a clamped-free beam condition. In the same beam condition, the nylon beam was used for static experiments. In the dynamic experiments, the mechanical vibration was monitored through CFBG-based sensors, while in the static experiments, the strain was monitored. For each case, the finite-element method (FEM) was applied to simulate the static and dynamic experiments to compare with the experimental results. The contribution of this work is the use of CFBGs for the simultaneous assessment of the force and strain distribution on the grating region. In addition, the vibration monitoring using the CFBG enabled the simultaneous static and dynamic assessment of a novel approach for structural monitoring using grating-based devices.

## 2. Materials and Methods

### 2.1. Simulations

**Figure 1:**
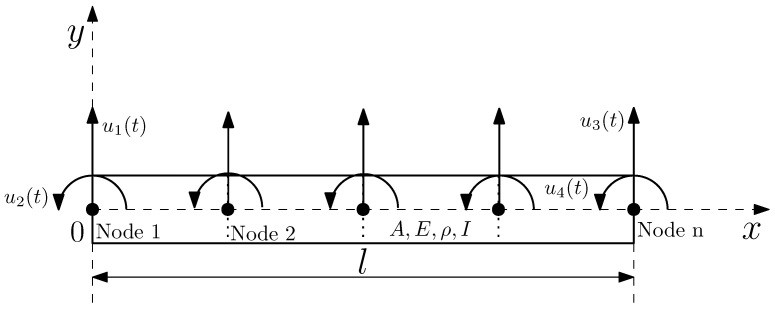
Euler–Bernoulli beam model.

FEM is a numerical method for modeling and solving boundary-value problems such as the frequency response of a loaded beam to study its mechanical vibration behavior with variational and interpolation methods [32]. In a discretization process, complex structures are divided into small parts called finite elements. There is an equation of motion for each element that can be easily solved or approximated. As a result, a linear combination of low-order polynomials is approximated using an assembly procedure, resulting in global mass and stiffness matrices that describe the mechanical vibration of the whole structure. An Euler–Bernoulli beam model is presented in Figure 1.

The coordinates used in the FEM model for the beam shown above are two linear coordinates, $u_{1}\left(t\right)$ and $u_{3}\left(t\right)$, and two rotational coordinates, $u_{2}\left(t\right)$ and $u_{4}\left(t\right)$. In this way, each node is modeled as having two degrees of freedom. The transverse static displacement must satisfy Equation (1) [32].

(1) $$\frac{\partial^{2}}{\partial x^{2}}\left[EI\frac{\partial^{2}u\left(x,t\right)}{\partial x^{2}}\right]=0$$

In Equation (1), *E* is Young’s modulus and *I* is the moment of inertia of the beam. For constant values of *E* and *I*, Equation (1) can be integrated to yield Equation (2).

(2) $$u\left(x,t\right)=c_{1}\left(t\right)x^{3}+c_{2}\left(t\right)x^{2}+c_{3}\left(t\right)x+c_{4}\left(t\right)$$

In Equation (2), $c_{i}\left(t\right)$ are constants of integration with respect to the spatial variable *x*. This equation is used to approximate the transverse displacement within the element used to discretize the geometry. The boundary conditions are shown in Equation (3).

(3) $$\begin{array}{cc} u\left(0,t\right)=u_{1}\left(t\right) & u_{x}\left(0,t\right)=u_{2}\left(t\right)\\ u\left(l,t\right)=u_{3}\left(t\right) & u_{x}\left(l,t\right)=u_{4}\left(t\right) \end{array}$$

In Equation (3), $u_{x}$ is the displacement derivative with respect to *x* (the rotational displacement). Combining Equation (3) and Equation (2) yields Equation (4).

(4) $$\begin{array}{c} c_{4}\left(t\right)=u_{1}\left(t\right)\;c_{3}\left(t\right)=u_{2}\left(t\right)\\ c_{2}\left(t\right)=\frac{1}{l^{2}}\left[3\left(u_{3}-u_{1}\right)-l\left(2u_{2}+u_{4}\right)\right]\\ c_{1}\left(t\right)=\frac{1}{l^{3}}\left[2\left(u_{1}-u_{3}\right)+l\left(u_{2}+u_{4}\right)\right] \end{array}$$

In Equation (4), *l* is the beam length. Substitution of Equation (4) into Equation (2) and rearranging terms yield Equation (5).

(5) $$\begin{array}{cc} u(x,t)= & \left[1-3\frac{x^{2}}{l^{2}}+2\frac{x^{3}}{l^{3}}\right]u_{1}\left(t\right)+l\left[\frac{x}{l}-2\frac{x^{2}}{l^{2}}+\frac{x^{3}}{l^{3}}\right]u_{2}\left(t\right)\\ \; & +\left[3\frac{x^{2}}{l^{2}}-2\frac{x^{3}}{l^{3}}\right]u_{3}\left(t\right)+l\left[-\frac{x^{2}}{l^{2}}+\frac{x^{3}}{l^{3}}\right]u_{4}\left(t\right) \end{array}$$

For the beam model, the mass matrix is calculated by Equation (6), where *A* is the cross-sectional area and ρ is the density of the beam.

(6) $$M=\frac{\rho Al}{420}\left[\begin{array}{cccc} 156 & 22l & 54 & -13l\\ 22l & 4l^{2} & 13l & -3l^{2}\\ 54 & 13l & 156 & -22l\\ -13l & -3l^{2} & -22l & 4l^{2} \end{array}\right]$$

The stiffness matrix is calculated by Equation (7).

(7) $$K=\frac{EI}{l^{3}}\left[\begin{array}{cccc} 12 & 6l & -12 & 6l\\ 6l & 4l^{2} & -6l & 2l^{2}\\ -12 & -6l & 12 & -6l\\ 6l & 2l^{2} & -6l & 4l^{2} \end{array}\right]$$

The transverse displacement for each element is described by the vector u(t), shown in Equation (8).

(8) $$u\left(t\right)=\left[\begin{array}{c} u_{1}\left(t\right)\\ u_{2}\left(t\right)\\ u_{3}\left(t\right)\\ u_{4}\left(t\right) \end{array}\right]$$

The strain energy for the beam can be factored into the form shown in Equation (9).

(9) $$V\left(t\right)=\frac{1}{2}u^{T}Ku$$

Finally, the acceleration for each element is calculated by Equation (10).

(10) $$M\ddot{u}\left(t\right)+Ku\left(t\right)=F\left(t\right)$$

Each nodal point acceleration in the geometry discretization is calculated using the above procedure. When no force is applied to a region, F(t) becomes zero. In this work, a model of a clamped-free beam was considered and an unbalanced load was applied at the free end of the beam. The unbalanced load is calculated by Equation (11), where $m_{0}$ is the unbalanced mass, *e* is the distance between the mass and the center of rotation, and $w_{r}$ is the rotation frequency.

(11) $$F\left(t\right)=m_{0}ew_{r}^{2}\sin\left(w_{r}t\right)$$

The FEM was performed using Ansys 2019 R3, in which the virtual model of the beam with the same dimensions as the real beam was used. Furthermore, the material chosen in Ansys has the same modulus of elasticity as Nylon 6.0, according to the structural element used to reproduce the experiment. In the static simulation, the support mode of the single-cantilever beam with load application on the free end was performed. The strain was generated as an output parameter of the software to evaluate the effect on the region in which the CFBG was positioned.

In order to perform the modal analysis, we followed the procedure outlined in [32]. The first step was to apply the Cholesky decomposition to the mass matrix *M*, obtaining $M^{-1/2}$. Then, the mass-normalized stiffness matrix is calculated by Equation (12).

(12) $$\tilde{K}=M^{-1/2}KM^{-1/2}$$

By solving the symmetric eigenvalue problem for $\tilde{K}$, we were able to determine the natural frequencies of the system. A modal analysis using Ansys 2019 R3 was combined with this method to provide a graphical representation of the vibration shapes.

In order to analyze the harmonic response of the beam, first, the displacement and acceleration were calculated by the FEM model described above. By applying the fast Fourier transformation (FFT), the displacement results were then used to examine the mechanical behavior in the frequency domain. This procedure was applied to each rotation velocity used in the dynamic tests for comparison with the experimental data.

### 2.2. Experimental Setup

Two beams were used in this work: the Nylon 6.0 and Pinus wood beams were used to perform the static and dynamic experiments, respectively. The Nylon 6.0 beam has a circular cross-section of 16 mm in diameter, whereas the Pinus wood beam has a rectangular cross-section of 101 mm × 11 mm. According to ISO 527 and ABNT MB26/53 standards, the Nylon 6.0 and the Pinus wood have an elastic modulus of 3200 MPa and 6463 MPa, respectively. For both experiments, the support mode of the single-cantilever beam was considered in which the static loads and unbalanced motor were positioned on the beam’s free end for the static and dynamic tests, respectively.

It is worth mentioning that the adopted reference (i.e., Position 0) is the anchored point of the beams. In addition, a chirped FBG of 25 mm in length (fabricated using the UV laser system, as reported in [33]) was attached to the upper surface of the Nylon 6.0 beam, being positioned between 101.0 cm and 103.5 cm of the length of the beam to evaluate the static behavior. Furthermore, a chirped FBG also was attached to the upper surface of the Pinus wood beam to evaluate its vibrational behavior under unbalanced load conditions.

**Figure 2:**
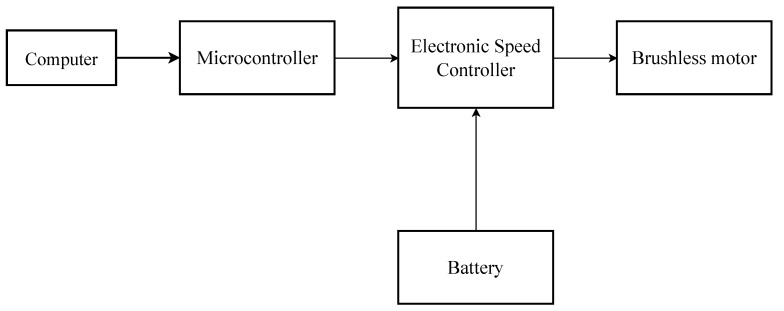
Motor assembly and control.

A brushless motor (Gartt, ML2212) with an unbalancing load was used to excite the Pinus wood beam through different dynamic loads. To operate the motor, we used a battery as the energy source, an electronic speed controller (ESC) (Hobbywing, skywalker 40 A) to change the rotation frequency, and a microcontroller connected to the computer to digitally control the rotation frequency as the input. The battery connected to the speed controller provides energy to accelerate the motor, whereas the speed controller connected to the microcontroller provides the settings to digitally control the rotation frequency by the computer. The pulse-width modulation (PWM) method was used for motor velocity control, in which the nominal voltage provided by the device is related to the activation and inactivation time of the output voltage. In this way, it was possible to change the rotation frequency of the motor, providing more than one condition to evaluate the vibrational behavior of the beam. For the motor unbalancing, an unbalanced propeller blade was connected to the rotor. Figure 2 presents the motor assembly and control performed in this work.

In the dynamic tests conducted with the Pinus beam, the Hyperion si155 (Luna Inc.) with a 5000 Hz data acquisition frequency and 1 pm resolution was used to measure the Bragg wavelength shift. During the static tests conducted with the Nylon beam, Micron Optics’ Static Optical Sensing Interrogator, Model SM125, with a data acquisition frequency of 2 Hz and a resolution of 1 pm, was used. The CFBG was inscribed in a photosensitive single-mode fiber (ThorLabs GF1AA) via the phase mask technique using a Nd:YAG laser with a central wavelength of 266 nm and a 8 ns pulse time (LOTIS TII LS-2137ULaser).

**Figure 3:**
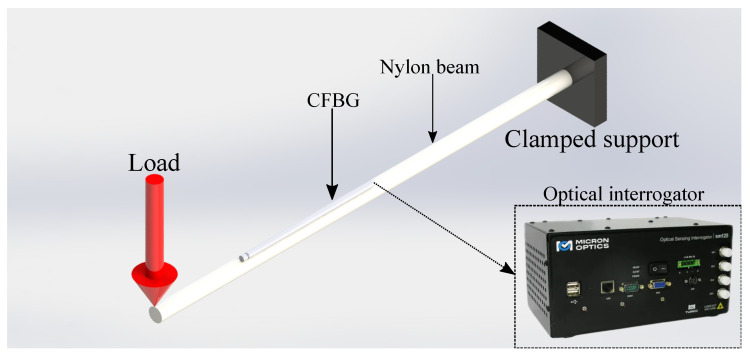
Schematic representation of the static test setup.

The static test setup is schematically shown in Figure 3. At the free end of the beam, weights ranging from 1 kg to 5 kg were applied in steps of 1 kg. An optical interrogator was used to measure the CFBG’s Bragg wavelength shift, as well as the reflected spectra acquisition for each load condition.

**Figure 4:**
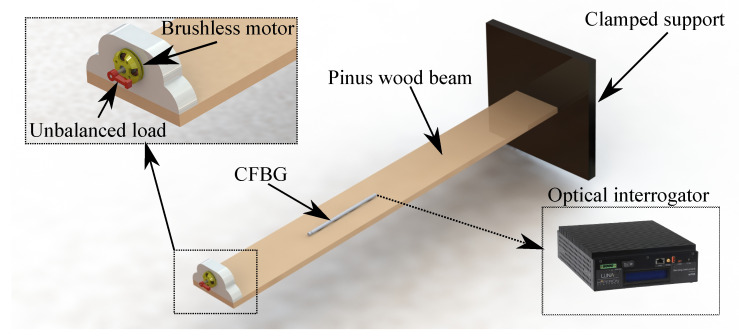
Schematic representation of the dynamic test setup.

The dynamic test setup is schematically shown in Figure 4. There was an unbalanced load applied to the brushless motor that was attached to the free end of the Pinus wood beam. The motor was operated at 16 Hz, 65 Hz, 101 Hz, and 131 Hz. To evaluate the mechanical vibration of the beam, an optical interrogator was used to measure the Bragg wavelength shift of the CFBG for each rotation velocity.

## 3. Results and Discussion

### 3.1. Simulation Results

**Figure 5:**
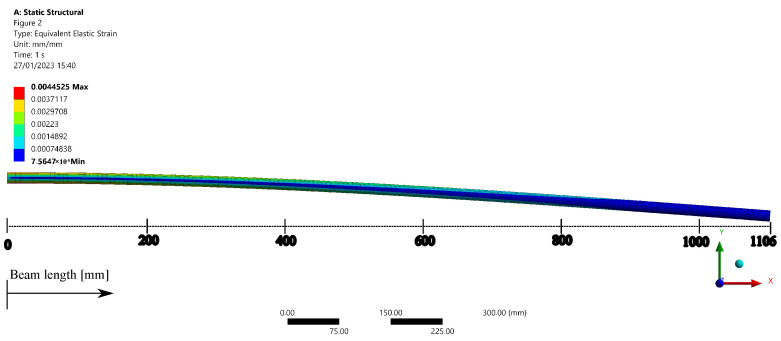
FEM simulation results of equivalent elastic strain on the static tests for a 50 N load applied at the free end of the beam (position at 1106 mm).

Figure 5 shows the equivalent elastic strain for the 10 N load applied on the free end of the beam, where it is possible to observe a higher strain on the regions close to the beam clamping position with a maximum elastic strain around 45μϵ. These results also indicate the shape (or elastic line) of the beam, which can also be experimentally estimated from the use of FBG arrays positioned along the beam [34].

**Figure 6:**
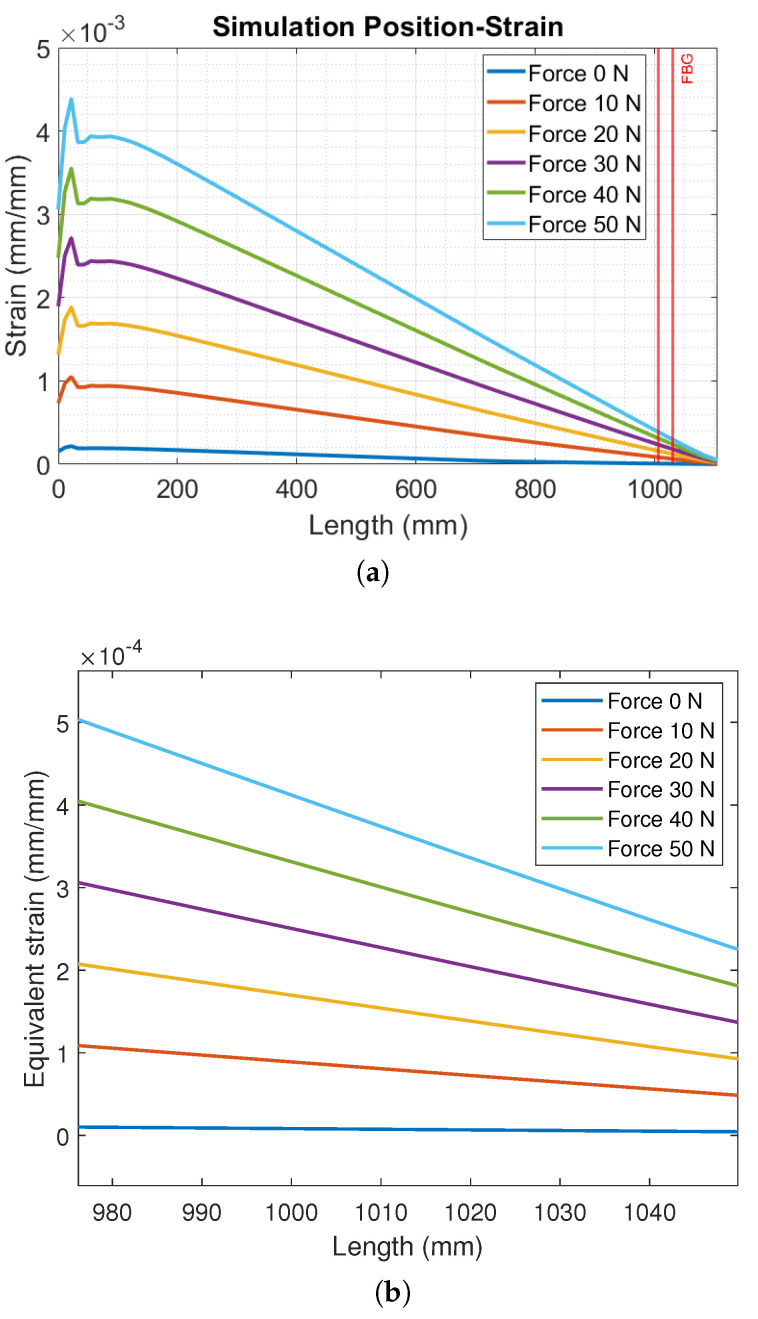
(**a**) Strain variation over the length of the virtual beam due to different loadings. (**b**) Strain variation in the FBG region for different loading conditions.

The simulation’s data enabled evaluating the strain variation over the length of the virtual beam, as presented by Figure 6a. The region in which the chirped FBG was positioned in relation to the real beam is represented by the span between the vertical lines in the graphic. In addition, Figure 6b shows the an approximated view to present a better view of the strain distribution along the CFBG region.

From the evaluation of the results in Figure 6a, it is worth noting that the strain peak was reached close to the anchored point, and after 150 mm of length, a linear behavior of the strain as a function of the length of the beam was observed for all magnitudes of the applied load. However, a greater load provoked a greater inclination in the linear region of the curves. By the simulation, it is also possible to note that the position in which the chirped FBG was attached suffered fewer variations of the strains compared to the region closer to the anchored point, requiring a higher sensitivity of the sensor. Regarding this, Figure 6b shows a closer view of the strain variation in the FBG region, where it is possible to observe different slopes on the curves. The highest spectral difference in the FBG response was obtained between sequential curves from a force of 0 N (slope of $7.8\times 10^{-8}$) to 10 N (slope of $8.2\times 10^{-7}$). In addition, forces of 20 N, 30 N, 40 N, and 50 N presented slopes of $1.6\times 10^{-6}$, $2.3\times 10^{-6}$, $3.0\times 10^{-6}$, and $3.8\times 10^{-6}$, respectively. This result can lead to the possibility of estimating the strain distribution in the FBG region using the CFBG spectral width variation with a linear relation between the applied force and the slope on the curves in Figure 6b.

**Figure 7:**
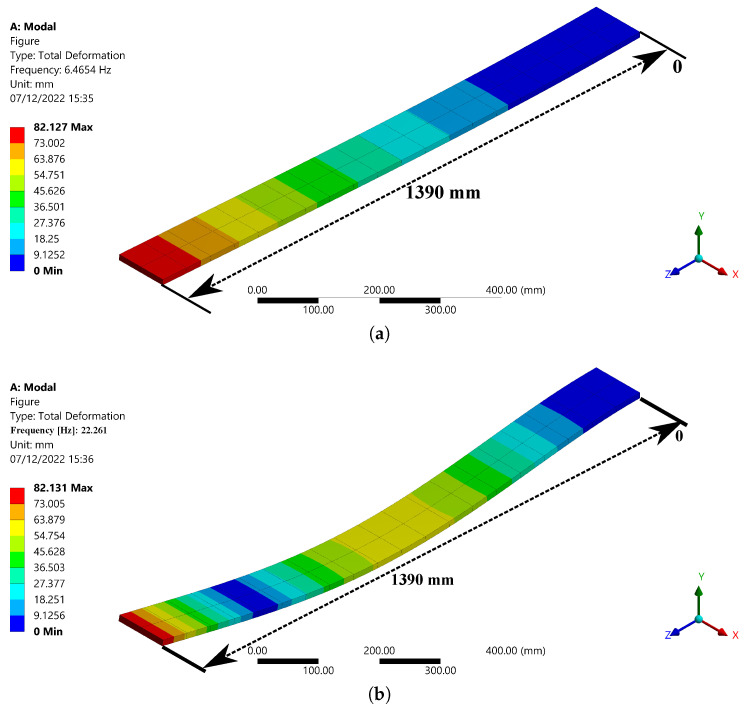

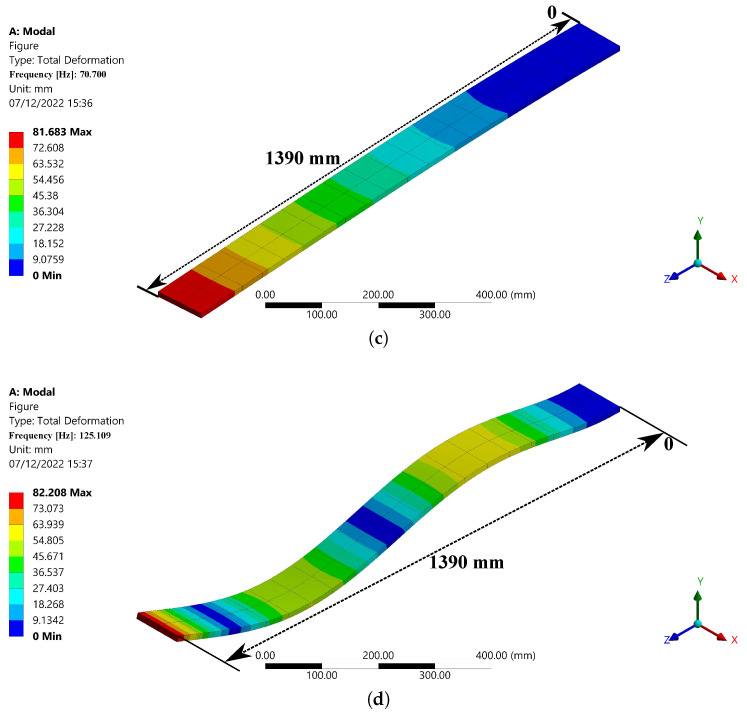
Vibration shape for (**a**) Mode 1, (**b**) Mode 2, (**c**) Mode 3, and (**d**) Mode 4.

The first four natural frequencies of the Pinus wood beam are shown in Table 1. To specify the beam’s safe range of excitation, these frequencies must be specified, as excitation frequencies near each natural frequency are expected to cause high-amplitude vibrations, which would compromise its structural integrity. The vibration shape for each natural frequency is shown in Figure 7. The vibration shape modes provide strategic information about the behavior of a structure at each natural frequency and the maximum displacement point. This information helps to identify which parts of the beam to embed CFBG sensors and enhance the measurements for low-intensity vibrations through heightened mechanical deformation applied to CFBG sensors.

**Figure 8:**
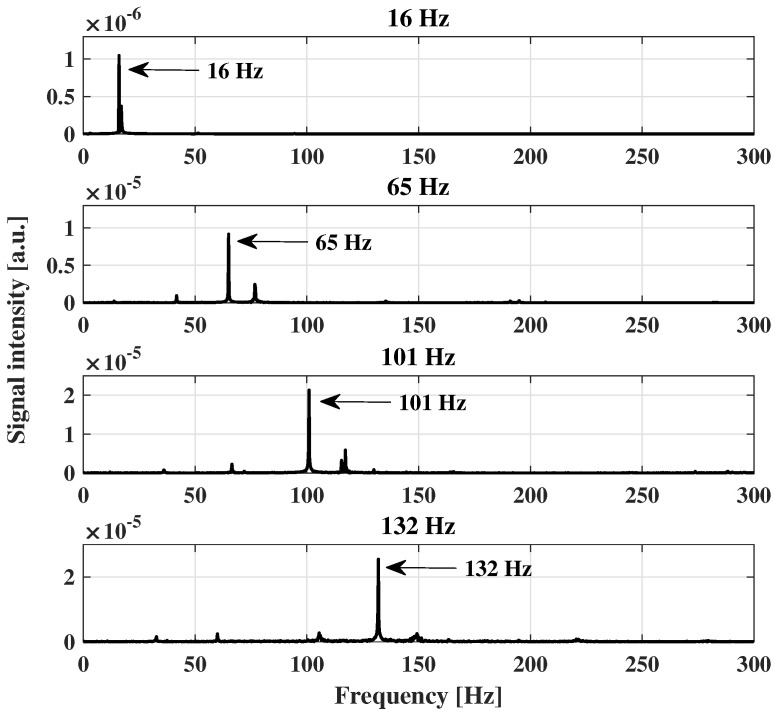
Harmonic response for each motor rotation velocity used in the dynamic FEM simulation.

The results of the harmonic response analysis are shown in Figure 8. The nominal rotation frequency of each case could be identified by the highest signal intensity in the frequency spectrum. As the unbalanced load is fixed to the brushless motor shaft and rotates at the same speed as the rotor, it increases the amplitude of the mechanical vibration at the nominal frequency. The frequency spectrum in each case is characteristic of an unbalanced rotor [35]. Since the FEM method only takes into account this effect, it ignores other nonlinear behavior caused by material anisotropy or assembly issues such as clearance and misalignment.

### 3.2. Experimental Results

**Figure 9:**
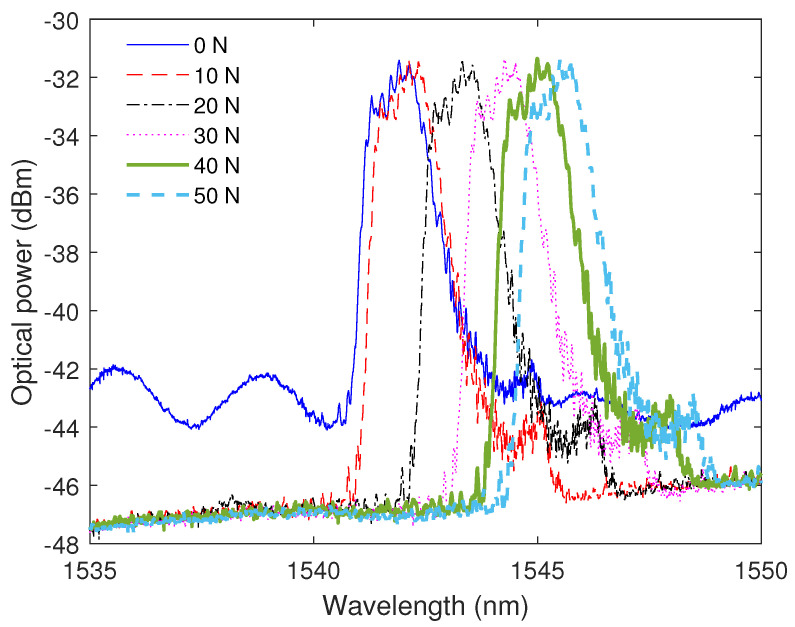
CFBG reflected spectra at different forces.

In both static and dynamic tests, the sensors system is subjected to a constant temperature, since the optical fiber sensors are sensitive to this parameter as well. Figure 9 shows the reflected spectra of the CFBG for each force condition of the static test. The results indicate a wavelength shift of the grating device as a function of the force. In addition, it is possible to observe that there is no significant variation of the optical power as a function of the force. Furthermore, the full-width at half-maximum (FWHM) of the spectrum of each force condition can also be related to the strain distribution along the grating region [21] with the additional possibility of using the combination of the FWHM and wavelength shift for temperature compensation approaches [36].

**Figure 10:**
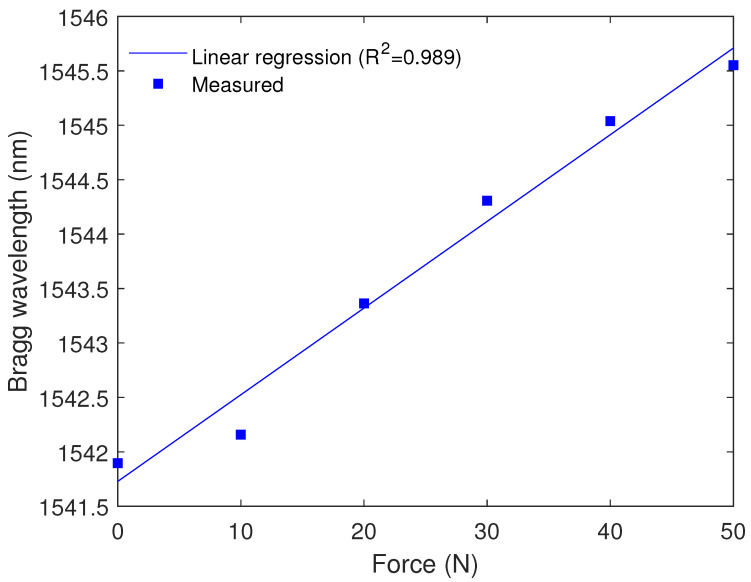
Bragg wavelength as a function of the force applied to the beam.

For the force assessment, the wavelength shift was analyzed as a function of the force, where the center wavelength was obtained from the middle point of the reflected spectrum. The results presented in Figure 10 show the linear trend on the sensor responses with a determination coefficient ($R^{2}$) of around 0.99. In addition, the sensor showed a sensitivity of 79.64 pm/N, for which, considering the spectral resolution of the optical interrogator (5 pm) and the standard deviation of the measurement (15 pm), it is possible to assume that the sensor can measure forces as small as 0.25 N.

**Figure 11:**
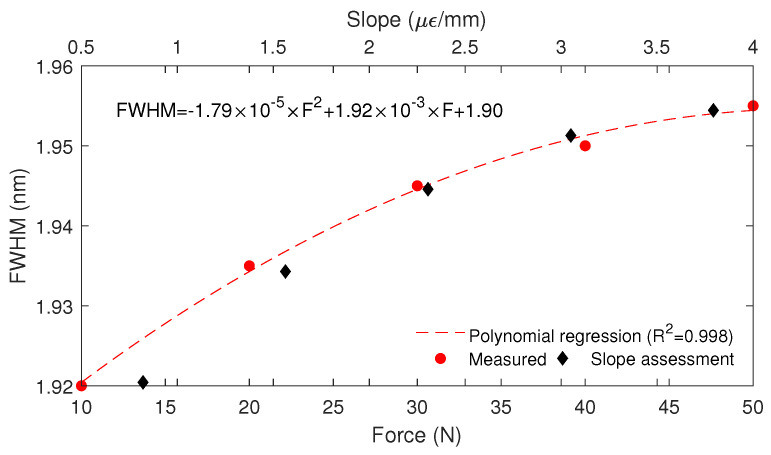
FWHM as a function of the force applied to the beam.

The FWHM analysis enabled the evaluation of the strain distribution on the beam. Regarding this, the linear variation of the strain in the FBG region was considered based on the strain simulations from the FEM static analysis. It is important to mention that the slope of the strain distribution along the CFBG region was proportional to the FWHM of the grating spectra, as presented in Figure 11. The $R^{2}$ of 0.998 between the measured data and the polynomial regression showed the high correlation and the feasibility of using the combination of the FWHM and Bragg wavelength shift for the force assessment. In addition, the results indicated a possible saturation trend for forces higher than 50 N, which can be related to the strain behavior of the material with a possible saturation at higher forces, as commonly occurs with different materials. Furthermore, the FWHM variation indicates the strain distribution along the CFBG, which can be used for the strain distribution assessment, as previously mentioned. In this context, the linear strain distribution was related to the slope of the linear response in the FBG region, as shown in Figure 6b. In this case, the slope estimation from the FWHM enabled the strain estimation along the CFBG region. Thus, from the slope in conjunction with the grating length, it is possible to estimate the strain variation along the CFBG, where the slope (in μϵ/mm) multiplied by the grating length resulted in the maximum strain variation in the FBG region. For this reason, the combination of the wavelength shift with the FWHM data resulted in the possibility of the simultaneous measurement of the force (or strain depending on the sensor characterization) and the strain distribution along the FBG region.

**Figure 12:**
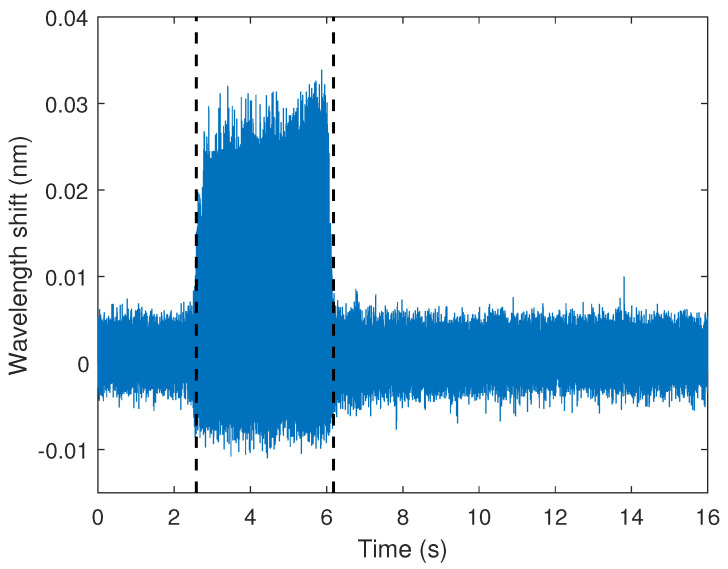
Wavelength shift as a function of the time for the vibration monitoring with the unbalanced motor activation. The dashed lines indicate the time of motor activation/deactivation.

In the dynamic tests using an unbalanced load on the motor, there was the vibration of the beam, which was transmitted to the optical fiber and led to the wavelength shift in the CFBG. In order to verify such a condition, Figure 12 shows the wavelength shift as a function of time for the motor at 1800 rpm, where it is possible to observe a higher variation of the wavelength shift when the unbalanced motor was activated. Considering the sensor sensitivity as a function of the force, there was a maximum force of around 0.5 N, which can be observed between the dashed lines in the sensor response, which represent the times of motor activation and deactivation. Such results indicate the suitability of the CFBG sensor for the analysis of vibration and acceleration on the structural element subjected to harmonic loads. It is also worth mentioning that the tests at different angular velocities of the motor led to similar maximum forces obtained in the wavelength shift, where the major difference between the curves was related to the period of the oscillatory wavelength shift.

**Figure 13:**
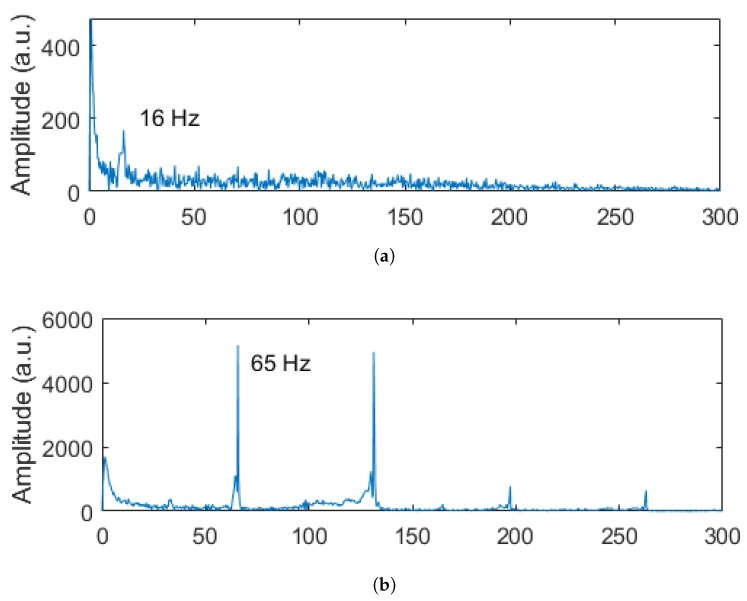

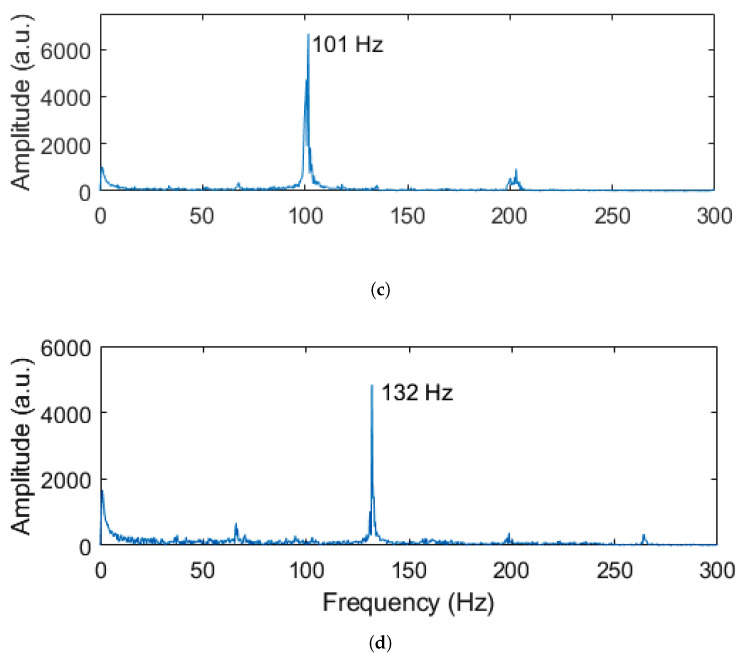
Frequency spectra of the sensors’ responses for different excitation frequencies of the motor: (**a**) 16 Hz, (**b**) 65 Hz, (**c**) 101 Hz, and (**d**) 132 Hz.

In order to evaluate the sensor response in the frequency domain for each condition, Figure 13 presents the frequency spectra of the wavelength shift at each angular velocity condition. The results indicated that the frequency at the maximum amplitude was related to the excitation frequency of the motor, where the highest amplitude was the excitation frequency of the motor in all analyzed cases. In addition, there were harmonics in the sensors’ responses, in which it is possible to observe the higher amplitude around 130 Hz in Figure 13b, where such a harmonic can be related to misalignment, as well as other transverse vibration conditions in the beam and the material anisotropy, especially considering the wood material.

In mechanical vibration analysis, harmonics are related to abnormal working conditions. Therefore, this parameter is strategic for structural health monitoring and fault diagnosis. For rotational loads, the appearance of the first harmonic (as seen at 65 Hz) is closely related to the mechanical looseness [35] of the internal parts of the rotor or some parts of the setup used in the clamped-free beam dynamic experiments. For higher frequencies (101 and 132 Hz), the mechanical vibration amplitudes are expected to be higher [37]. Moreover, the unbalanced load intensifies the amplitude of the frequency response at nominal excitation. As a result, the second harmonic in those cases was much smaller, and the signal was filtered along with the noise. At 16 Hz, only the nominal frequency was identified due to the unbalanced load effect. In this case, the second harmonic signal was at the same level as the noise and could not be identified. Some filtering techniques could be applied at 16 Hz to reduce the noise and improve the signal-to-noise ratio. There is, however, a trade-off associated with these techniques, and some data can be lost as a result. Thus, for a better diagnosis of failures in structures, different frequencies must be analyzed, as performed in this work.

A cross-sensitivity between the temperature and mechanical deformation occurs in CFBG-based sensors. However, both dynamic and static experiments were conducted in a temperature-controlled environment. Temperature variation was smaller during these tests than the mechanical excitation (mechanical vibration for dynamic experiments and load application on the free end of the beam for static experiments). Consequently, in these cases, the Bragg wavelength shift can be attributed primarily to mechanical excitation, and thermal effects can be ignored. In the case of higher variations in the temperature, there was a wavelength shift on the Bragg wavelength, which can be compensated using an additional sensor isolated from the strain for the compensation of the temperature-induced wavelength shift. For the dynamic experiments, the frequency of harmonic excitation caused by the unbalanced rotor located at the free end of the beam was significantly higher than the variation in room temperature, which supports neglecting the thermal effects.

## 4. Conclusions

This paper presented the development and analysis of a CFBG for the static and dynamic monitoring of beams. Nylon and wooden beams were used for the experiments, where their responses were numerically evaluated through FEM analysis. In this case, the vibration modes, the natural frequencies, and the strain distribution on the beam subjected to a static load were numerically analyzed, where a linear strain distribution was found on the beam, especially in the region at which the CFBG was installed. Then, the experimental results using the CFBG responses showed a high correlation between the force applied on the beam and the wavelength shift ($R^{2}=0.99$) with a potential resolution of 0.25 N. In addition, the strain distribution was evaluated using the FWHM of the CFBG, which presented an exponential trend as a function of the force and on the slope of the linear strain distribution along the fiber ($R^{2}\;\mathrm{higher}\;\mathrm{than}\;0.99$ for all analyzed cases). In the dynamic tests, the CFBG was able to detect the excitation frequencies through the strain/force variation (estimated from the wavelength shift) transmitted from the beam to the optical fiber, where the additional frequencies and harmonics were related to backlash, transverse deformation, and secondary effects due to the material anisotropy. It is also worth noting that the strain variation along the FBG region can be detected with higher resolution by positioning additional CFBGs at the regions of higher strain on the beam. Therefore, the proposed sensor system was able to provide the simultaneous assessment of different static and dynamic parameters using only one CFBG, where the multiplexing capabilities of such a sensor approach also made it suitable for the installation of additional CFBGs along the structural element. For this reason, future works include the heterogeneous sensor system for the simultaneous axial and transverse assessment of the strain/vibration in different materials using CBFG arrays. Moreover, future works will include tests of the proposed sensors in smaller force ranges and in different materials.

## Figures and Tables

**Table 1:** Natural frequencies of the Pinus wood beam.

Mode	Frequency (Hz)
1	18.0619
2	107.199
3	322.655
4	593.427

## Data Availability

The data presented in this study are available upon request from the corresponding author.
